# Supplementary material for: Identification of photoperiod-induced specific miRNAs in the adrenal glands of Sunite sheep (Ovis aries)
Source: Front Vet Sci. 2022 Jul 22;9:888207. doi: 10.3389/fvets.2022.888207 (PMC9354845; doi:10.3389/fvets.2022.888207)
Supplement: Supplementary file 1 [file Data_Sheet_1.ZIP › Supplementary file/miRDeep2 file.zip!/miRDeep2 file/Novel_Structure/LP42A3/Novel_253.pdf]

Provisional ID	:	NC_019462.2_8788
Score total	:	1.6
Score for star read(s)	:	-1.3
Score for read counts	:	0
Score for mfe	:	1.3
Score for randfold	:	1.6
Score for cons. seed	:	
Total read count	:	128
Mature read count	:	128
Loop read count	:	0
Star read count	:	0

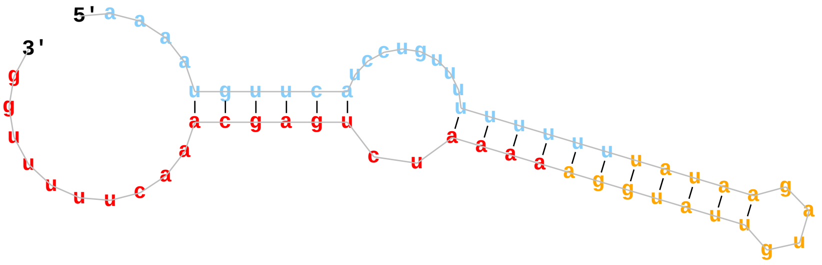

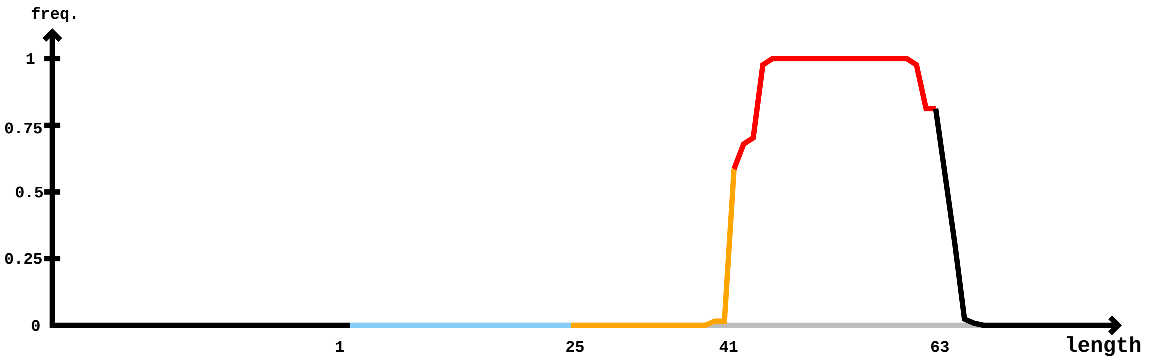

Star Mature

```
5'- uuuaaacaguaugauguauugguuuggccaaaauguucauccuguuuuuuuuuuauaagauguuauggaaaaaucugagcaaacuuuuuggccaauccaguauuucagu   -3'    exp
    ...........(((.(((((((.((((((((((.(((.....((((((((((((((((....)))))))))))...)))))))).)))))))))).))))))).)))..   reads     mm     sample
    ...................................................................gaaaaaCcugagcaaacuuuu.....................   2         1      seq
    .....................................................................aaaaCcugagcaaacuuuu.....................   1         1      seq
    .....................................................................aaaaucugaAcaaacuuuuu....................   20        1      seq
    .....................................................................aaaaucugaAcaaacuuuuugg..................   51        1      seq
    .....................................................................aaaaucugaAcaaacuuuuuggc.................   1         1      seq
    ......................................................................aaaucugagcaaacuuuuu....................   1         0      seq
    ......................................................................aaaucugaAcaaacuuuuugg..................   11        1      seq
    .......................................................................aaucugaAcaaacuuuuuggc.................   1         1      seq
    .......................................................................aaucugaAcaaacuuuuuggcc................   2         1      seq
    ........................................................................aucugaAcaaacuuuuuggcc................   34        1      seq
    ........................................................................aucugaAcaaacuuuuuggccaa..............   1         1      seq
    .........................................................................ucugaAcaaacuuuuuggc.................   1         1      seq
    .........................................................................ucugaAcaaacuuuuuggcca...............   2         1      seq
```
